# Effects of Phenosanic Acid in Rat Seizure Models

**DOI:** 10.3390/ijms26125668

**Published:** 2025-06-13

**Authors:** Victor A. Aniol, Natalia A. Lazareva, Yulia V. Moiseeva, Olga A. Nedogreeva, Margarita R. Novikova, Pavel A. Kostryukov, Mikhail V. Onufriev, Natalia V. Gulyaeva

**Affiliations:** 1 Laboratory of Functional Biochemistry of the Nervous System, Institute of Higher Nervous Activity and Neurophysiology, Russian Academy of Sciences, 117485 Moscow, Russia; aniviktor@yandex.ru (V.A.A.);; 2 Research and Clinical Center for Neuropsychiatry of Moscow Healthcare Department, 115419 Moscow, Russia

**Keywords:** epilepsy, pentylenetetrazole, lithium–pilocarpine epilepsy model, phenosanic acid, dibufelon, neurogenesis, neurodegeneration, hippocampus, corticosterone, free radical oxidation

## Abstract

Oxidative stress and membrane damage are believed to be principally involved in the pathogenesis of epilepsy. This study aimed to assess the effects of phenosanic acid (PA), an antioxidant and membrane protector, in acute pentylenetetrazole and chronic lithium–pilocarpine seizure models in male Wistar rats. PA was administered acutely (ip, 120 mg/kg BW ip, or 240 mg/kg BW per os) or chronically (80 mg/kg BW/day per os). Indices of free radical oxidation, the hypothalamo–pituitary–adrenocortical axis, and the nitrergic system were assessed in blood and brain regions. Morphological analysis of the hippocampus was performed in the lithium–pilocarpine model. PA exerted an acute anti-seizure effect in the pentylenetetrazole model. In the lithium–pilocarpine model, acute PA treatment decreased the death rate and corticosterone levels in the neocortex and brainstem. In contrast, the level of free radical oxidation products reacting with thiobarbituric acid declined in the brain stem in response to chronic PA treatment. In the lithium–pilocarpine model, the neuronal density in the dentate gyrus was elevated, and the proliferating cell nuclear antigen positive (PCNA+) cell counts in the subgranular zone did not differ between groups. Doublecortin positive (DCX+) cell count was significantly increased after chronic PA treatment. PA-induced reduction in mortality in the lithium–pilocarpine epilepsy model may be partially mediated by decreasing the lipid peroxidation and corticosterone levels in different brain regions. Chronic PA treatment may affect adult hippocampal neurogenesis by either prolonging the action of factors that increase neurogenesis after status epilepticus or by slowing down the neuronal differentiation rate. These data suggest that PA may be a disease-modifying AED able to hamper epileptogenesis.

## 1. Introduction

Epilepsy is a chronic brain disease, characterized by recurrent seizures, that affects about 70 million people worldwide [1]. Among the main problems in epileptology that have not yet been completely solved is the absence of drugs that could in a truly aetiotropic manner, prevent not only the seizure occurrence but the formation of an epileptogenic area itself, as well as control the drug resistance [2]. Existing antiepileptic drugs (AEDs), which are used in the clinic, mainly aim at reducing the frequency of seizures and prolonging the inter-seizure intervals. Often, this aim cannot be achieved by a single AED; therefore, polytherapy is needed. Basic AEDs can also have a number of undesirable side effects [3]. For example, they may cause and/or exacerbate comorbid pathology and have a toxic effect on the central nervous system. The pro-oxidative effect of some existing AEDs may even increase convulsive readiness due to increased excitability of neurons and/or induction of neuronal damage, followed by the development of functional tolerance to anticonvulsant therapy. Altogether, this may be the reason for the use of potential antioxidants and neuroprotectors in the complex therapy of epilepsy, which may increase the viability of neurons and neuronal plasticity, as well as regulate the metabolic activity of neurons [4].

Phenozanic acid (PA) is a phenol-derivative synthetic antioxidant. It stabilizes neuronal membranes by inhibiting the peroxidation process and changing the lipid composition of the cell membranes [5]. PA is the active substance of the original antiepileptic drug Dibufelon registered in Russia and used as a part of combined therapy in patients with partial epileptic seizures with or without secondary generalization [6].

For a deeper understanding of PA effects and its more effective clinical use, a study of the molecular and cellular mechanisms of its action on the brain using valid animal models is vital. One of the most reliable rodent models of epileptogenesis is the pilocarpine-induced status epilepticus (SE) model, which was first developed by the Turski group over 40 years ago [7]. The advantage of this model is the manifestation of spontaneous seizures, which makes it an adequate model of epilepsy in humans. Pilocarpine administration (in some cases preceded by lithium chloride to potentiate the convulsive effect) leads to a rapid development of SE, which, in turn, triggers the process of epileptogenesis in the following several weeks [8]. During this latent period, neuropathological alterations, including neuroinflammation, degeneration, and death of neurons in the hippocampus and amygdala, as well as changes in adult hippocampal neurogenesis, occur in the brain. All these alterations are believed to form the structural basis for a further decrease in the seizure threshold and the appearance of spontaneous seizures. Similar changes (neuroinflammation, gliosis, neurodegeneration, aberrant neurogenesis) were also revealed in brain samples of patients with drug-resistant epilepsy [9]. The available data suggest that the lithium–pilocarpine model is a valid approach for studying the mechanisms of epileptogenesis in animals and testing the antiepileptogenic activity of drugs in preclinical experiments. The existence of a latent period for the development of biochemical and structural changes in the brain makes it possible to study the effects of drugs at the stage when the development of the pathological neural network occurs, as well as to evaluate the ability of these drugs to prevent epileptogenesis, not just seizures.

The reported antioxidant, membrane-stabilizing, and adaptogenic effects of PA [10,11,12,13] suggest its potential neuroprotective effectiveness during epileptogenesis. In this regard, we aimed to study the anticonvulsant effect in the acute pentylenetetrazole seizure model and potential antiepileptogenic effects of PA in the latent period of lithium–pilocarpine SE and assess biochemical (intensity of free radical processes and the level of adrenal hormones) and morphological (neuronal degeneration and cell death in the hippocampus as well as some markers of neurogenesis) alterations induced by seizures and PA. Two models were used in accordance with different aspects of PA action studied: the acute PTZ model was chosen for its simplicity and validity in estimating the seizure threshold, whereas the lithium–pilocarpine model was used to examine the possible prevention of epileptic circuitry development by the PA during the latent period after the SE.

## 2. Results

### 2.1. Acute Pentylenetetrazole (PTZ) Model

**Figure 1:**
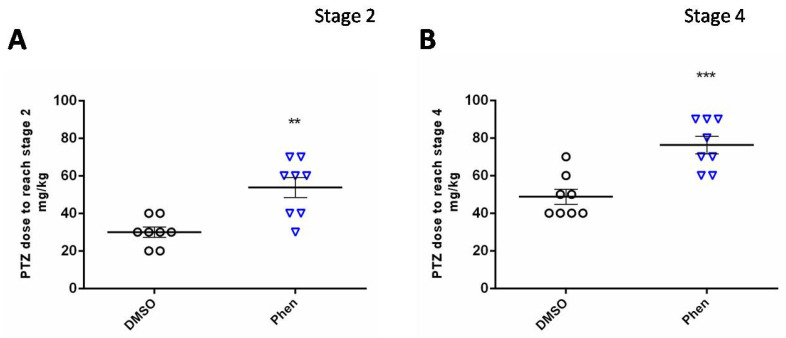
Effect of acute PA administration on the severity of PTZ-induced seizures. PTZ concentrations necessary to reach Stage 2 (**A**) and Stage 4 (**B**) are shown. **—*p* < 0.01; ***—*p* < 0.005; Mann–Whitney U-test. DMSO—vehicle group; Phen—group acutely administered with PA. Data are presented as M ± SEM.

A single i.p. administration of PA resulted in decreased sensitivity of rats to the acute convulsive effect of PTZ. The amount of PTZ necessary to induce clonic seizures (Stage 2) or generalized clonic–tonic convulsions with falling (Stage 4) was significantly increased in rats treated with PA (Figure 1).

However, the acute anti-convulsive effect of PA may not be sufficient alone for the prevention of epileptogenesis. To find out whether PA may prevent the development of pathological alterations associated with chronic epilepsy, we analyzed the effect of PA in a model of pilocarpine-induced SE.

### 2.2. Chronic Lithium–Pilocarpine Model

In this model, PA was administered orally after pilocarpine-induced SE either chronically during a 5-week period starting from the second day after the SE, followed by one week of washout (to assess the effect on the development of epilepsy-like alterations), or once one hour before the sacrifice, 6 weeks after the SE (to assess the effect of a single dose on the epileptic brain).

#### 2.2.1. Seizures

**Figure 2:**
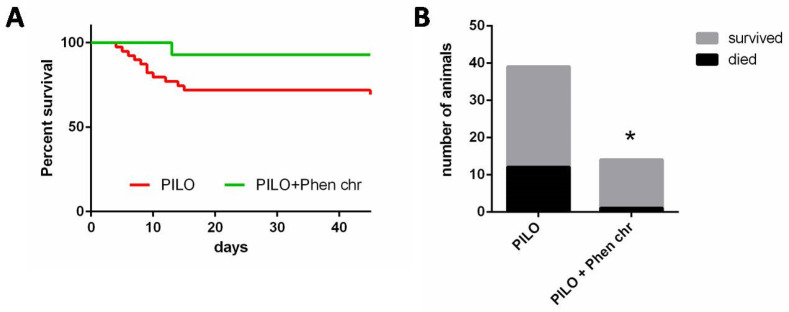
Effect of chronic PA administration on the survival of animals. (**A**) Survival curve timeline in pilocarpine (PILO) post-SE animals with no treatment (80 mg/kg, orally; Phen chr). (**B**)—Number of animals that died or survived in both groups during the period of treatment (starting from the second day post-SE). *—*p* < 0.05; Chi-square test.

Pilocarpine administration induced the development of SE (Stage 5, repetitive generalized seizures with falling, lasting for no less than 1 h) in a majority of rats (49 of 68 rats, 72%), while the cumulative dose of pilocarpine necessary to reach this stage was 21 ± 2 mg/kg in these rats. The remaining 19 animals demonstrated only Stage 3 seizures even after reaching the maximal dose of pilocarpine (40 mg/kg) and failed to develop the SE. All animals without SE survived for the following 6 weeks. However, of the 49 animals that experienced the SE, more than half (28 rats, or 57%) died during the following two weeks, and about half of them died in the first two days after the SE (15 rats). After the start of PA therapy on day 2 post-SE, only one rat died in the chronic PA treatment group, in contrast to 12 rats that died during the consecutive days in the group treated with the vehicle (Figure 2, *p* < 0.05; Chi-square test).

#### 2.2.2. Biochemical Assays

First, we compared all biochemical indices in the sub-groups according to the different sensitivities of the seizures induced by pilocarpine (resistant rats, which did not develop SE after pilocarpine injection and sensitive rats which developed full-grade SE). Since no difference was found between these subgroups, we combined them for further analysis.

**Figure 3:**
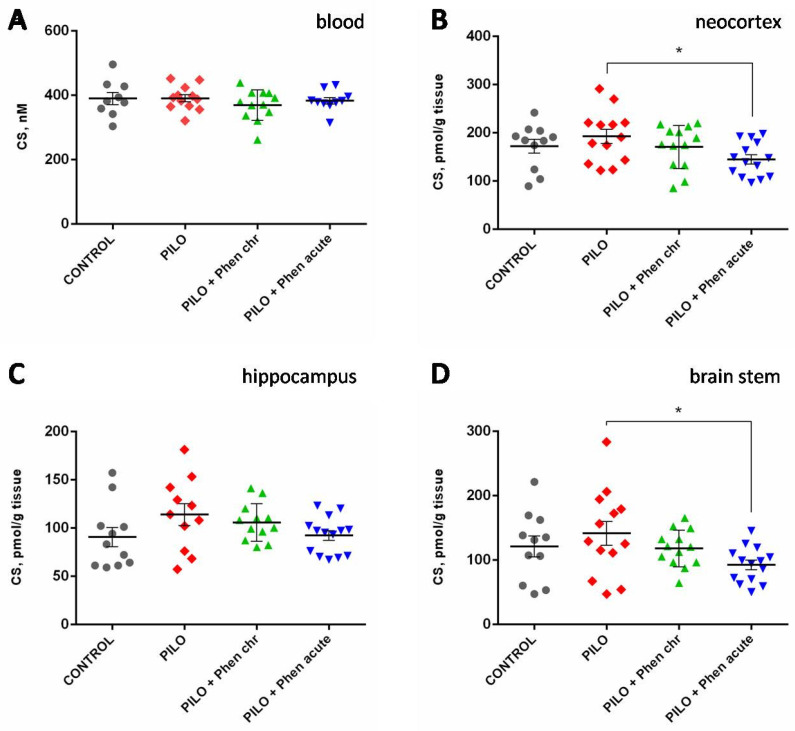
Corticosterone level in blood plasma (**A**) and different brain regions: neocortex (**B**), hippocampus (**C**), and brain stem (**D**) of rats. CONTROL—control group; PILO—pilocarpine group; Phen chr—group chronically treated with PA; Phen acute—group acutely treated with PA. *—*p* < 0.05; Mann–Whitney U-test. Data are presented as M ± SEM.

The level of corticosterone did not change significantly in rats that experienced pilocarpine-induced SE 6 weeks before as compared to control animals. Chronic PA treatment did not change the corticosterone level either (Figure 3). Neither pilocarpine nor PA affected the levels of corticosterone (Figure 3A) or ACTH in the blood (Figure S1). However, acute PA treatment one hour before the sacrificing decreased the level of corticosterone in the neocortex and brain stem of rats experiencing pilocarpine-induced seizures (*p* < 0.05, Mann–Whitney U-test, Figure 3B,D), whereas in the hippocampus, this effect was less significant, showing just a trend (*p* < 0.1, Mann–Whitney U-test).

The total amount of nitrates and nitrites (NOx) was decreased in the blood of rats 6 weeks after pilocarpine SE (*p* < 0.05, Mann–Whitney U-test), while the groups with either chronic or acute PA treatment did not differ significantly from respective control levels (Figure S2). In the brain regions, neither pilocarpine nor subsequent PA treatment (chronic or acute) affected the level of NOx. The difference between control and pilocarpine-treated rats in the blood could be rather attributed to NO_3_ but not NO_2_, since NO_3_ demonstrated the same pattern of changes, whereas NO_2_ did not respond to either treatment (Figures S3 and S4).

**Figure 4:**
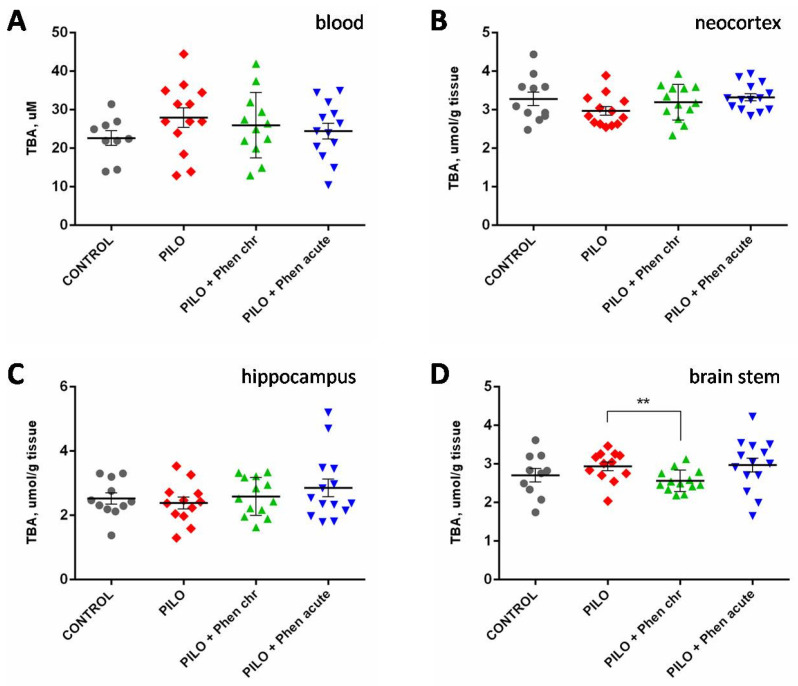
TBARS concentration in blood plasma (**A**) and different brain regions: neocortex (**B**), hippocampus (**C**), and brain stem (**D**) of rats. CONTROL—control group; PILO—pilocarpine group; Phen chr—group chronically treated with PA; Phen acute—group acutely treated with PA. **—*p* < 0.01; Mann–Whitney U-test. Data are presented as M ± SEM.

SOD activity also demonstrated no significant changes in response to pilocarpine and/or PA treatment in the blood as well as in the brain (Figure S5). The level of free radical oxidation products reacting with thiobarbituric acid (TBARS) decreased in the brain stem in response to chronic PA treatment as compared to untreated pilocarpine group. No significant changes could be revealed in the neocortical or hippocampal tissue, as well as in the blood (Figure 4).

#### 2.2.3. Morphological Analysis

Despite the obvious severe neuronal degeneration in CA1 and CA3 hippocampal areas of some rats that experienced pilocarpine-induced SE (Figures S6 and S7), most animals demonstrated only minor changes to the structure of these areas as compared to the control group. Notably, one animal from the pilocarpine group demonstrated hippocampal sclerosis. On the whole, there was no statistically significant decrease in the number of neurons in CA1 and CA3 areas after pilocarpine-induced SE (Figures S6 and S7).

**Figure 5:**
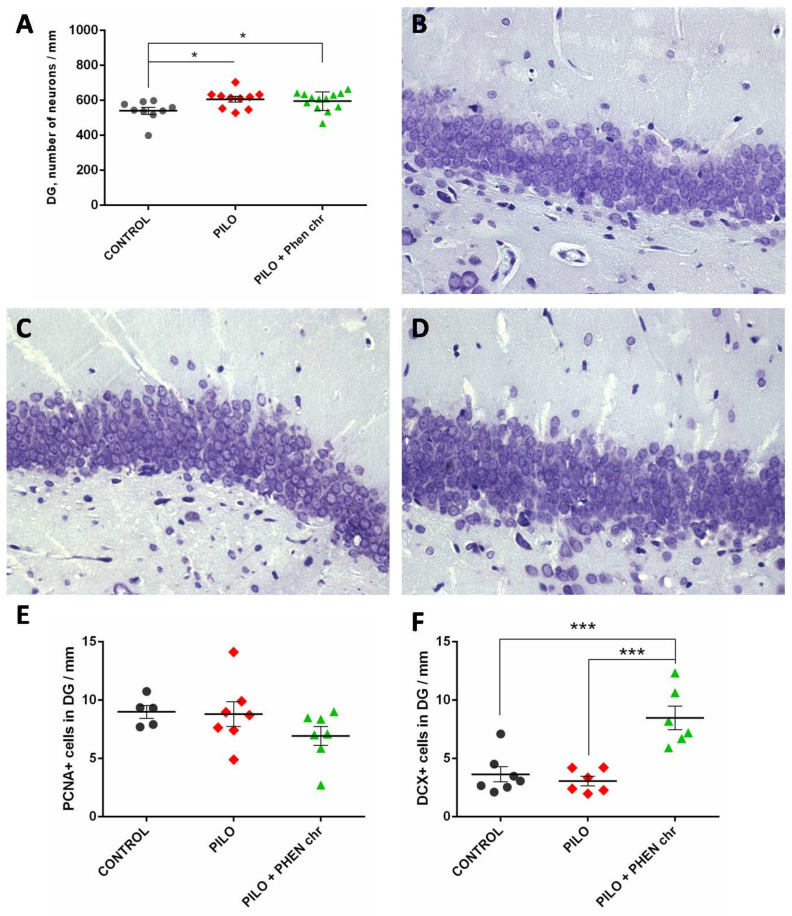
Morphological changes in the DG induced by pilocarpine and PA. (**A**) Total number of neurons in the granular layer of the DG. (**B**–**D**): representative microimages of rats from the control group (**B**), pilocarpine SE group (**C**), and PA-treated pilocarpine-SE group (**D**). (**E**,**F**): The number of PCNA+ cells (**E**) and DCX+ cells (**F**) in the DG. CONTROL—control group; PILO—pilocarpine group; PILO + Phen chr—group chronically treated with PA. *—*p* < 0.05; ***—*p* < 0.005; Mann–Whitney U-test. Data are presented as M ± SEM. ×400.

An interesting phenomenon was revealed in the DG, where the number of neurons was elevated in rats which experienced pilocarpine-induced SE, no matter whether they were later treated with PA or not (*p* < 0.05, Mann–Whitney U-test, Figure 5A–D). PCNA+ cell count in the subgranular zone (SGZ) of the DG revealed no significant difference between the groups (Kruskal–Wallis test *p* > 0.1), suggesting that proliferation of neural progenitor cells either was not affected by pilocarpine-induced SE and PA treatment or did change but already returned to baseline by the end of sixth week of observation (Figure 5E). The latter hypothesis may be supported by further results of DCX+ cell count (Figure 5F). The number of DCX+ cells was significantly increased in the group with chronic PA treatment as compared to both the control group and post-SE group treated with the vehicle (*p* < 0.005, Mann–Whitney U-test).

## 3. Discussion

PA and its soluble salts (specifically, potassium phenosane) were shown to possess antioxidant and membranotropic properties in different experimental models in vitro and in vivo [11,12,13,14,15,16,17]. Beneficial effects of PA derivatives on the functioning of the brain and autonomic nervous system have been reported in preclinical studies. In a rat model of chronic emotional painful stress, administration of the potassium salt of PA prevented autonomic dysfunction, heart hypertrophy oxidative stress in the brain [10]. Using the KM rats susceptible to audiogenic seizures, Fedotova et al. showed beneficial effects of potassium salt of PA on cognitive functions [18]. PA was previously shown to attenuate the severity of seizures in different rodent models. Antiradical mechanism of the protective action of PA derivatives in epileptiform seizures in rats and their subsequent death from brain hemorrhage was confirmed [19]. PA administered alone or in combination with the valproic acid, a widely used AED, reduced the generalization of convulsive foci of epileptic activity and prevented the formation of a stable epileptic system in a model of focal cobalt-induced epilepsy [20]. Importantly, valproic acid, though possessing pronounced anticonvulsant properties, did not slow down the development of the epileptic system. Clinical use of PA, the active substance of the AED Dibufelon, showed that the addition of PA (Dibufelon) to base AED therapy as a second or third AED had beneficial effects on reducing the frequency and severity of epileptic seizures, as well as on comorbid affective and cognitive disorders, thus increasing the quality of life of patients with epilepsy [6,21]. It was suggested that targeting oxidative stress by PA may attenuate epileptogenesis in patients with epilepsy [22].

In the present study, we have confirmed an acute anti-convulsive effect of PA in the model of PTZ-induced seizures in rats. However, besides the anticonvulsive effect, we tried to find whether PA possesses an antiepileptogenic potential, the ability to prevent progression of epilepsy and development of drug resistance. Therefore, we tested PA in a chronic pilocarpine-induced SE model, a well-described and widely used model of epileptogenesis [7,23]. Pilocarpine is an M1 muscarinic acetylcholine receptor agonist that promotes continuous excitatory activity, resulting in brain tissue damage and spontaneous seizures [24]. Administration of pilocarpine results in progressive development of convulsive seizures from minimal external manifestations, such as oral–facial automatism, salivation, blinking, twitching of vibrissae, and yawning, through repeated attacks of clonic waves, which last for 90–150 min, to limbic motor seizures with intense salivation, standing on the hind limbs, clonus of the upper limbs, and falling. Such convulsive episodes occur every 5–15 min, with the maximum frequency taking place after 1–2 h. Pilocarpine induces the development of an epileptic status (SE) in about 60% of rats [25]; in a while, the animals may fall into a postictal coma for 1–2 days. Body weight usually slightly decreases after seizures (by 10–20%) but typically recovers to baseline values within a week [23]. Remarkable alterations in SE induction and mortality rates may exist among different rodent strains [26]. In addition, even the rats of the same strain may also differ by their seizure predisposition if obtained from different breeding locations or purchased at different times [27].

In our study, more than half of the animals successfully developed the SE. After the SE, the administration of PA allowed us to decrease the mortality rate of rats during the latent period. A possible mechanism of decreased mortality may be provided by attenuated lipid oxidation, as assessed by decreased TBARS levels in the homogenates of the brainstem, where most nuclei and brain structures controlling the vital functions reside.

Other mechanisms involved in the development of epilepsy and seizure-associated brain damage are glucocorticoid-dependent events [1,28]. Though in some cases, anti-inflammatory and immunosuppressive effects of glucocorticoids may prevent epileptogenesis [29], glucocorticoids have been reported to induce hippocampal hyperexcitability [30] and increase overall epileptiform activity [31]. Moreover, glucocorticoids are elevated for weeks following SE in animal models [32,33] and therefore could exert lasting negative consequences. Pharmacological blockade of glucocorticoid receptors in rodents could decrease the alterations in the hippocampus after the SE [33]. Here, we have demonstrated that acute administration of PA could decrease the level of corticosterone in some brain regions (neocortex and brain stem, with less prominent effects in the hippocampus). Chronic administration of PA was followed by a one-week withdrawal period; therefore, changes in the corticosterone level in this group may not be seen. However, since acute administration exerted a corticosterone-lowering effect, it should be supposed that chronic administration also functioned in this way, at least during the acute post-administration period. Though the nitric oxide system is tightly involved in the regulation of brain and immune mechanisms, in particular in epileptogenesis and seizures [34], we were unable to find significant changes in nitrate and nitrite levels in blood and brain regions induced by pilocarpine or PA. The only exception was a decrease in NO_3_ level in the blood after pilocarpine-induced SE, whereas chronic and acute PA groups did not differ from controls, suggesting some compensatory effect.

In the hippocampus, the damage to the principal cells of CA1 and CA3 regions was not statistically significant after the SE, despite selected animals that demonstrated the neuronal loss. Unexpectedly, SE induced a significant increase in the number of granule neurons in the DG, possibly reflecting the increased neurogenesis after the seizures. Adult neurogenesis occurs throughout the entire life in the DG of the hippocampus in most mammalian species, including humans [35], though its existence in adult humans still remains debatable [36,37,38]. Neural progenitor cells proliferate at the border between the granular cell layer and the underlying polymorphic layer (hilus) of the DG. It has been demonstrated that many cells born in the DG become granular neurons, but some of them migrate into the hilus and/or give rise to differentiated glial and endothelial cells [39]. In rodents, the differentiation of newly born cells into granular neurons takes roughly 30 days [40]. The rate of adult neurogenesis is generally increased after acute seizures of any origin, then slowly returns to the basal level during the following days or weeks. This has been shown in the models of SE induced by pilocarpine [41,42,43] or kainate [44,45], as well as in many kindling models [46,47,48,49,50]. In our study, we did not detect any increase in the level of ongoing proliferation in the DG assessed by PCNA+ cell count, thus supposing that the period of increased proliferation, if it did occur in our experiments, had already ended 6 weeks post-SE. However, the number of DCX+ cells in the DG was increased in animals treated with PA chronically, suggesting an ability of PA to affect certain stages of adult neurogenesis. Without additional experiments, it is hard to interpret this finding. Indeed, three possible mechanisms may be disputed in this regard. First, PA may prolong the period of increased neurogenesis after the SE, thus making some cells born during this period still visible 6 weeks after the SE. The same changes might appear in case PA prolongs the time period of neuronal differentiation. Finally, PA may affect not seizure-induced, but the basal level of neurogenesis, acting independently of pilocarpine-induced SE.

This study has several limitations. First of all, only male rats were used in the experiment, thus neglecting potential dependence of PTZ and pilocarpin-induced epileptic seizures as well as of PA effects on sex. Indeed, sex differences in the effectiveness of the AEDs, as well as the potential side effects in relation to reproductive function and pregnancy, may be of great importance in the case of chronically administered medications such as AEDs. However, the primary goal of the current study was to establish the possible anti-convulsive and anti-epileptogenic effects per se, leaving an investigation of sex differences an important objective for further studies. Therefore, males were chosen to minimize the animal groups and avoid the effect of the estrus cycle in the chronic experiment. Second, the reason for the PA-induced increase in DCX+ cells in the DG and the mechanisms of this phenomenon, suggesting an influence of PA on neurogenesis, remain obscure, and confirming one of the put-forward hypotheses remains a goal of further investigations. Another obvious limitation common to all animal models with high mortality is potential survivorship bias [51].

## 4. Materials and Methods

### 4.1. Animals

The experiments were carried out on male Wistar rats (supplied by Andreevka Branch of the Scientific Center for Biomedical Technologies of the Federal Medical and Biological Agency, Moscow Region, Russia). The experimental animals were kept in individual plastic cages (5–6 animals per cage) in the colony room with a 12 h light–dark cycle (light phase 8:00–20:00) and free access to food and water. All procedures were performed in accordance with the ARRIVE guidelines, the U.K. Animals (Scientific Procedures) Act 1986 and its associated guidelines, and the EU Directive 2010/63/EU for animal experiments. The Ethical Commission of the Institute of Higher Nervous Activity and Neurophysiology RAS approved our protocol (protocol # 3, 24 June 2021).

### 4.2. Seizure Models

#### 4.2.1. Acute PTZ-Model

To assess the acute anti-convulsant effect of PA, we applied a model of PTZ-induced convulsions. Adult male Wistar rats (250 g, *n* = 16) were used in this acute experiment. One hour before seizure induction, one half of the animals (*n* = 8) received a single i.p. injection of PA (240 mg/kg) dissolved in DMSO, and another half received the same volume of pure DMSO (1 mL/kg). One hour later, the rats were transferred into observation cages and received i.p. injections of PTZ, starting from a dose of 20 mg/kg with additional consecutive injections of 10 mg/kg every 5 min until reaching stage 4 according to the Racine scale (generalized clonic–tonic convulsions with falling). The cumulative dose of PTZ necessary to induce seizures was used as an index of seizure threshold.

#### 4.2.2. Chronic Lithium–Pilocarpine Model

Young male Wistar rats (180–200 g at the beginning of the experiment, *n* = 79) were used for the analysis of the PA influence on epileptogenesis in the lithium–pilocarpine SE model. Rats were randomly divided into four groups.

1. Control group (*n* = 11): Rats of this group were not subjected to lithium–pilocarpine seizures and received a vehicle (peanut butter) per os throughout the whole experiment.
2. Pilocarpine SE group (*n* = 23): These rats were subjected to a lithium–pilocarpine SE model and received a vehicle (peanut butter) per os throughout the whole experiment.
3. Pilocarpine SE group with chronic PA administration (*n* = 21): Rats of this group were subjected to lithium–pilocarpine SE, similarly to animals of the previous group, but received PA in the peanut butter per os (80 mg/kg daily) for 5 weeks after the SE, followed by a one-week period of vehicle administration (during week 6).
4. Pilocarpine SE group with acute PA administration (*n* = 24): Rats of this group were subjected to lithium–pilocarpine SE, similarly to animals of the two previous groups, but received a vehicle (peanut butter) per os throughout the whole experiment except for the last day, when PA (120 mg/kg) was administered.

On the day preceding the pilocarpine administration, the animals received an i.p. injection of LiCl solution at a dose of 100 mg/kg. On the next day, 1 h before pilocarpine administration, the animals were subjected to a blockade of peripheral muscarinic acetylcholine receptors by administration of methyl scopolamine bromide (1 mg/kg, i.p. in isotonic saline, 1 mL/kg). One hour later, pilocarpine administration started. Pilocarpine was injected i.p. at a dose of 5 mg/kg at 30 min intervals until reaching the total dose of 40 mg/kg or until manifestation of generalized clonic–tonic convulsive seizures (stages 4 or 5). If seizures developed, animals were injected with xylazine to relieve muscle convulsions (2.5 mg/kg; i.p. in isotonic saline). An hour after reaching stage 4 or 5, seizures were stopped by diazepam (10 mg/kg, i.p. in 50% DMSO, 4 mL/kg). After the cessation of motor convulsions and the onset of sleep, the animals were rehydrated by s.c. administration of 3 mL of isotonic saline with 5% glucose. Later, after waking up, the animals were offered water orally. After the final awakening, the animals were returned to the vivarium in their home cages. During the next two days, the animals that exhibited seizures received supportive s.c. injections of isotonic saline with 5% glucose. After the end of supportive therapy, on day 2 post-SE, the animals started receiving drug therapy (PA or vehicle) corresponding to their group distribution. These treatments lasted for 6 weeks after the pilocarpine-induced SE.

The poor water solubility of PA made it problematic to properly dose the substance for oral administration in the form of a water suspension. Therefore, PA was mixed with peanut butter at a dose of 80 mg/mL for chronic and 120 mg/mL for acute administration, and pure peanut butter was used as a control substance. The fresh mixture was made each day before the administration. The substances were placed in graduated syringes, which were used for dosed oral administration (in a volume of 1 mL/kg).

One hour after the last treatment, animals were terminally anesthetized with an i.p. injection of chloral hydrate (700 mg/kg) and decapitated. After decapitation, the brains were removed from the skull and divided into halves along the midline. Then, a randomly selected half was placed in a 10% formalin solution, and the neocortex, hippocampus, and medulla oblongata from the other half were isolated on ice, frozen in liquid nitrogen, and stored at −80 °C until use. Also, blood from the cervical vessels was collected after decapitation. After the blood was separated into phases at room temperature, it was centrifuged at 1500× *g* at a temperature of 4 °C for 15 min; then, the supernatant (blood serum) was taken and frozen at −80 °C.

### 4.3. Biochemical Assays

The isolated brain regions were homogenized in a Potter’s homogenizer using a standard phosphate-salt buffer. Aliquots were selected from the resulting homogenate, and the remaining homogenate was centrifuged at 13,000× *g* at 40 °C for 30 min to obtain a soluble protein fraction (supernatant), which was aliquoted and stored at −80 °C before biochemical analyses.

To measure the level of corticosterone in blood serum and homogenates of rat brain regions, a Corticosterone ELISA Kit (DRG, Marburg, Germany) was used for competitive ELISA, which allowed for detection of both free and transport protein-bound corticosterone. The blood level of ACTH was determined using the ACTH Extraction Free EIA ELISA kit (Phoenix Pharmaceuticals, Burlingame, CA, USA) according to the manufacturer’s instructions.

The blood serum and the homogenates of different brain regions were used for measuring different biochemical indices: free radical oxidation products reacting with 2-thiobarbituric acid (TBARS), superoxide dismutase activity (SOD), total amount of nitrates and nitrites (NOx), as well as nitrates (NO_3_) and nitrites (NO_2_) separately. For these analyses commercially available kits were used according to the manufacturer’s instructions: TBARS Assay Kit, SOD Assay Kit, and Nitrate/Nitrite Colorimetric Assay Kit for blood serum and the more sensitive Nitrate/Nitrite Fluorometric Assay Kit for different brain regions, all kits obtained from Cayman Chemical (Ann Arbor, MI, USA).

### 4.4. Brain Morphological Analysis

The brain halves assigned for morphological examination were stored in 70° ethanol before use. They were cut into 2 mm thick frontally oriented blocks. These blocks were placed into histological cassettes and processed according to the following protocol: 70° ethanol, 30 min; 96° ethanol, 1 h; isopropanol, 1 h, overnight in a new portion; xylene, 1 h, then 6 h in a new portion; xylene: paraffin 1:1 mixture, overnight at 58 °C; paraffin, 6 h at 58 °C, then overnight in a new portion at 58 °C; embedding in a fresh portion of paraffin.

On the next day, after paraffin embedding, the blocks were cut into 5 μm thick frontal sections using a rotation microtome (CUT 5062, Slee, Nieder-Olm, Germany). The slices were mounted onto gelatin-covered slides, stained with Cresyl violet, and coverslipped. Photomicrographs of the hippocampus (CA1 and CA3 areas at ×200 magnification, and dentate gyrus (DG) at ×400 magnification) were taken using a Leica DM 5000B microscope and the Leica Application Systems v. 3.6.0 software package (Leica Microsystems GmbH, Wetzlar, Germany). Five to ten images were taken from each of three consecutive sections of the hippocampus located 2 mm apart. Neurons were counted on the images obtained from the pyramidal (in CA1 and CA3 areas) and granular layers (in the DG). In addition, the length of the analyzed hippocampal layer within each image was measured for subsequent normalization of the obtained neuronal numbers by the length of the counting area. All calculations were performed manually in the ImageJ 1.50 g program (NIH, Bethesda, MD, USA). For each region (CA1, CA3, or DG), the final values were averaged over all photos and all sections for each animal.

For the total counting of proliferating cell nuclear antigen positive (PCNA+) and doublecortin positive (Dcx+) cells, we used an indirect immunoperoxidase staining. For each staining, three sections located 2 mm apart containing the hippocampus (a total of 6 sections for each staining) were taken. All sections were stained simultaneously for each antigen. The sections were deparaffinized in two portions of xylene and rehydrated in decreasing grade alcohols. Then, sections were rinsed in distilled water and 0.01 M phosphate-buffered saline (PBS). Endogenous peroxidases were quenched by 15 min incubation in 3% H_2_O_2_ in PBS, followed by triple rinse in PBS, after which the sections were blocked with 5% normal goat serum in PBS with 0.3% Triton X−100 for 1 h. Antibodies were dissolved in the same blocking solution and applied for one night at +4 °C (mouse monoclonal anti-PCNA 1:1000, Santa Cruz Biotechnology, Dallas, TX, USA; rabbit polyclonal anti-Dcx, 1:1500, Abcam, Cambridge, UK). On the next day, sections were thoroughly rinsed in PBS and incubated with secondary antibodies in the blocking solution for 2 h (goat anti-mouse IgG conjugated with biotin, 1:800, Jackson Immunoresearch, West Grove, PA, USA, or goat anti-rabbit IgG conjugated with biotin, 1:800, Sigma-Aldrich, St Louis, MO, USA). Sections were again rinsed in PBST (PBS with Tween−20) and then incubated with avidin-peroxidase complex (ABC Vectastain kit, Vector Laboratories, Burlingame, CA, USA; 1:100 in PBS) for 1 h. After that, sections were washed in PBS, and the peroxidase reaction was visualized with 3,3′-diaminobenzidine (DAB, Sigmafast kit; Sigma-Aldrich, St Louis, MO, USA) until the development of staining, controlled under the low-power binocular microscope (3–5 min). Finally, sections were thoroughly washed in PBS and coverslipped.

PCNA+ and DCX+ cells were then counted manually at ×400 magnification throughout the whole length of the DG on the section and divided by the length of the DG to obtain the number of cells per mm.

### 4.5. Statistical Analysis

The data were presented as M ± S.E.M. with individual values. A normality check was performed using the Kolmogorov–Smirnov test. Between-group comparisons were made using the Mann–Whitney U-test. Mortality was compared via the Chi-square test. The difference was regarded as significant at *p* < 0.05.

## 5. Conclusions

Altogether, we can conclude that PA exerts an acute anti-seizure effect in the PTZ convulsion model and prevents animal death in lithium–pilocarpine post-SE model. The latter is possibly achieved by decreasing the lipid peroxidation or corticosterone level in different brain regions. Also, chronic PA treatment affects adult hippocampal neurogenesis after the lithium–pilocarpine SE, either by prolonging the action of factors increasing neurogenesis after the SE or by slowing down the rate of neuronal differentiation, both looking like increased number of neuroblasts and immature neurons 6 weeks after the SE.

## Data Availability

The raw data supporting the conclusions of this article will be made available by the authors on request.

## Supplementary Materials

The following supporting information can be downloaded at https://www.mdpi.com/article/10.3390/ijms26125668/s1.

## Abbreviations

The following abbreviations are used in this manuscript:

ACTH	Adrenocorticotropic hormone
AEDs	Antiepileptic drugs
DCX+ cells	Doublecortin positive cells
DG	Dentate gyrus
NOx	Total amount of nitrates and nitrites
PA	Phenosanic acid
PBS	Phosphate-buffered saline
PCNA+ cells	Proliferating cell nuclear antigen positive cells
PTZ	Pentylenetetrazole
SE	Status epilepticus
SGZ	Subgranular zone
SOD	Superoxide dismutase
TBARPS	Substances reacting with 2-thiobarbituric acid
