# Supplementary material for: Effects of Phenosanic Acid in Rat Seizure Models
Source: Int J Mol Sci. 2025 Jun 13;26(12):5668. doi: 10.3390/ijms26125668 (PMC12193637; doi:10.3390/ijms26125668)
Supplement: Supplementary file 1 [file ijms-26-05668-s001.zip › ijms-3678989-supplementary.pdf]

SUPPLEMENTARY MATERIAL

to "Effects of phenosanic acid in rat seizure models" by Victor A. Aniol, Natalia A. Lazareva, Yulia V. Moiseeva, Olga A. Nedogreeva, Margarita R. Novikova, Pavel A. Kostryukov, Mikhail V. Onufriev, Natalia V. Gulyaeva

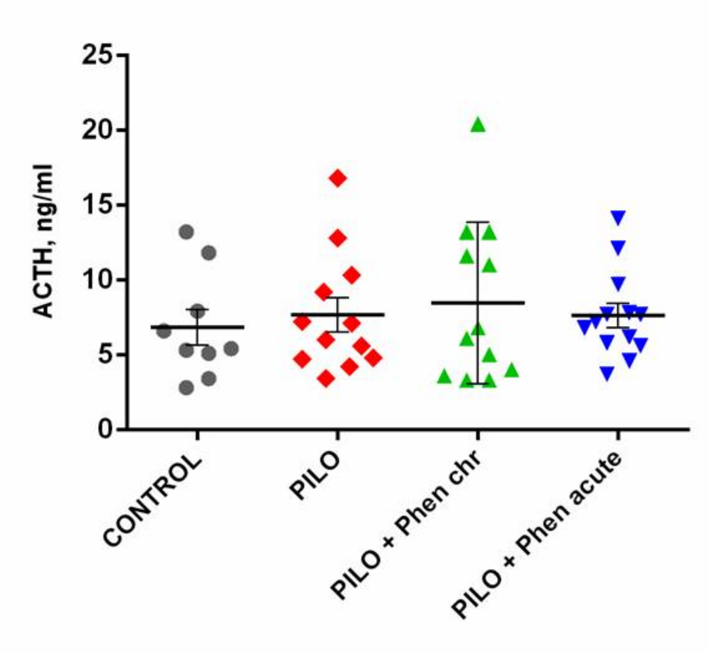

Figure S1. ACTH level in blood plasma. CONTROL - control group; PILO - pilocarpine group; Phen chr - group chronically treated with PA; Phen acute - group acutely treated with PA. Data are presented as M±SEM.

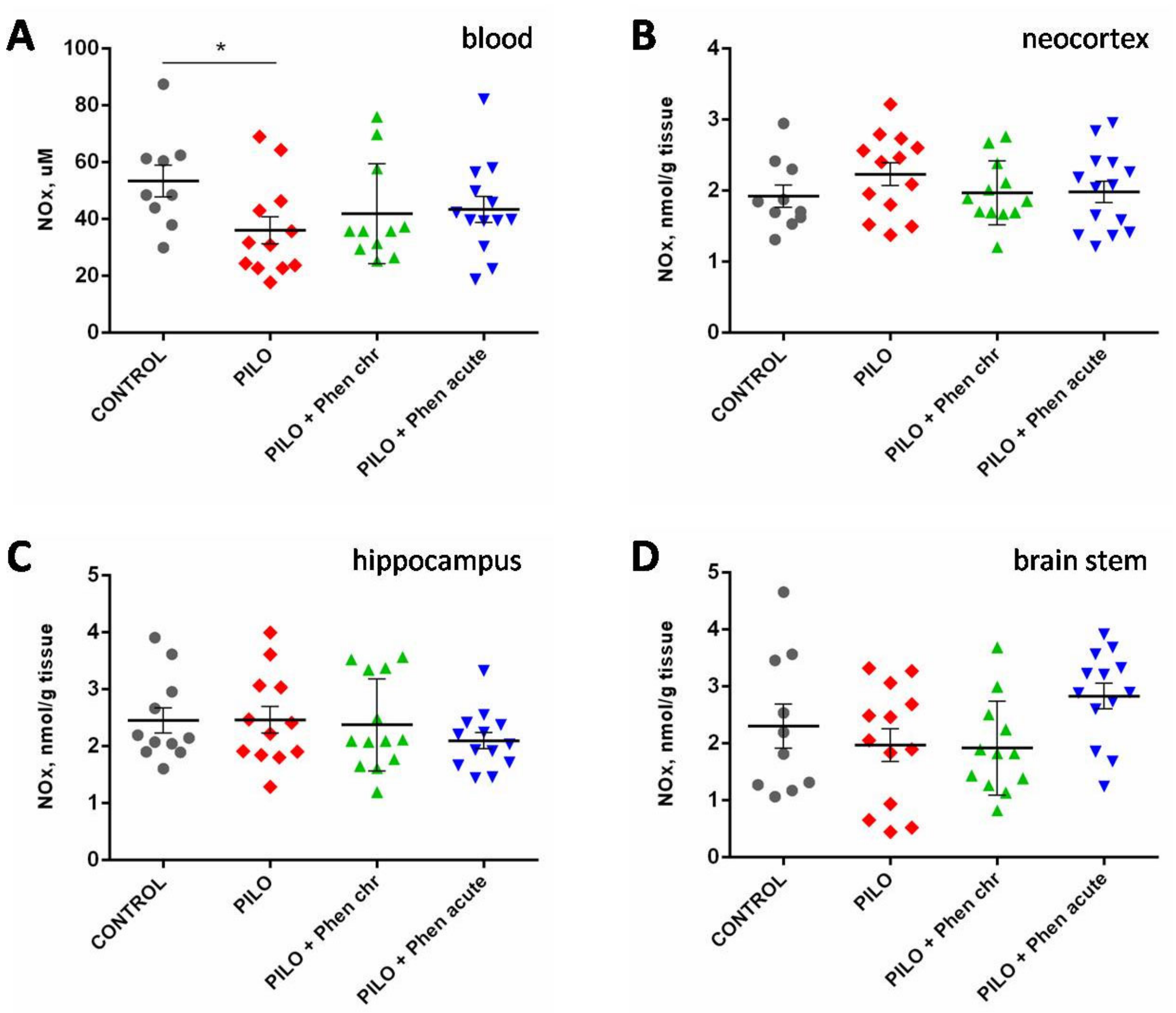

Figure S2. NOx level in blood plasma (A) and different brain regions: neocortex (B), hippocampus (C), and brain stem (D) of rats. CONTROL - control group; PILO - pilocarpine group; Phen chr - group chronically treated with PA; Phen acute - group acutely treated with PA. * - P<0.05; Mann-Whitney U-test. * - P<0.05; Mann-Whitney U-test. Data are presented as M±SEM.

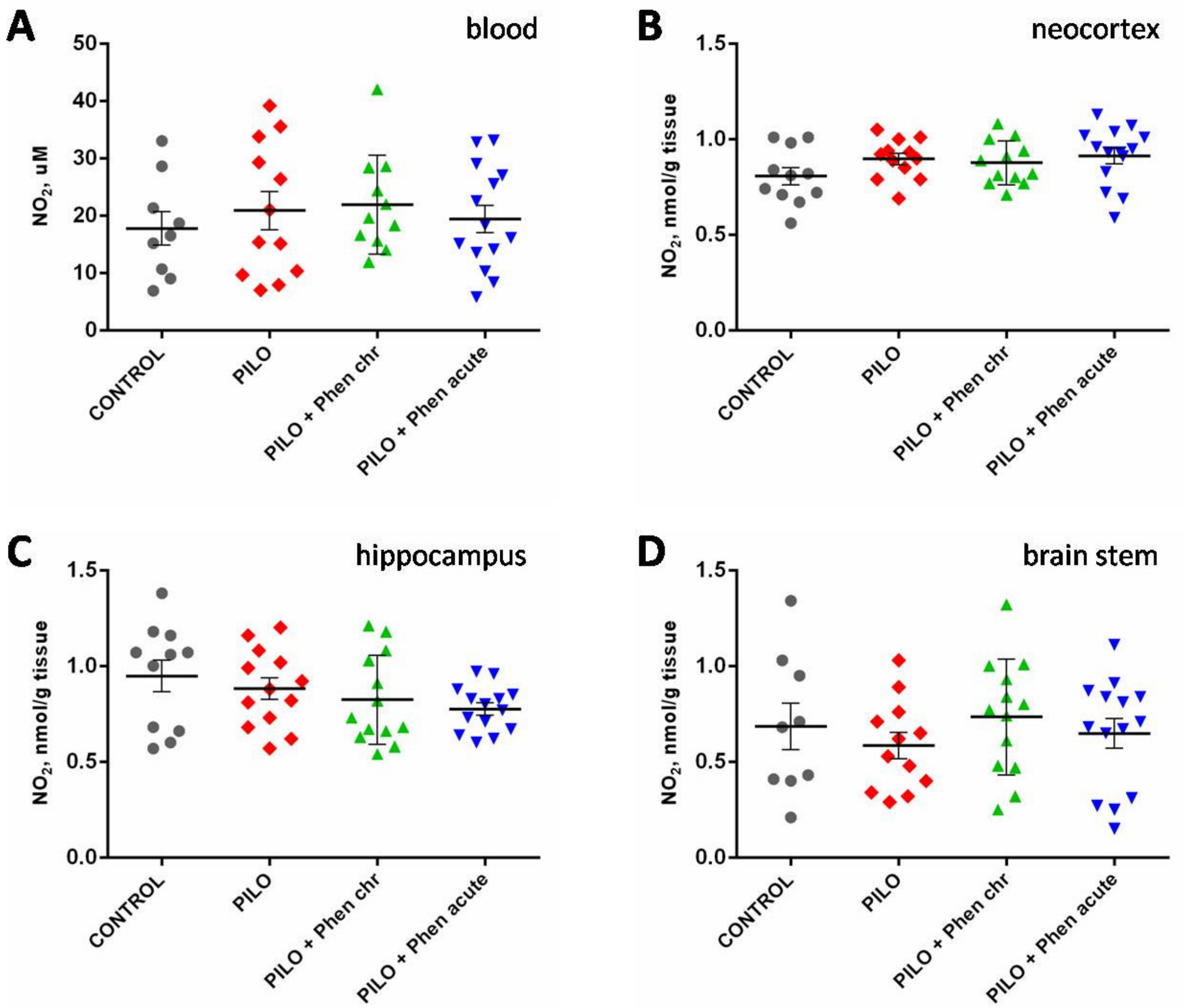

Figure S3. NO2 level in blood plasma (A) and different brain regions: neocortex (B), hippocampus (C), and brain stem (D) of rats. CONTROL - control group; PILO - pilocarpine group; Phen chr - group chronically treated with PA; Phen acute - group acutely treated with PA. Data are presented as M±SEM.

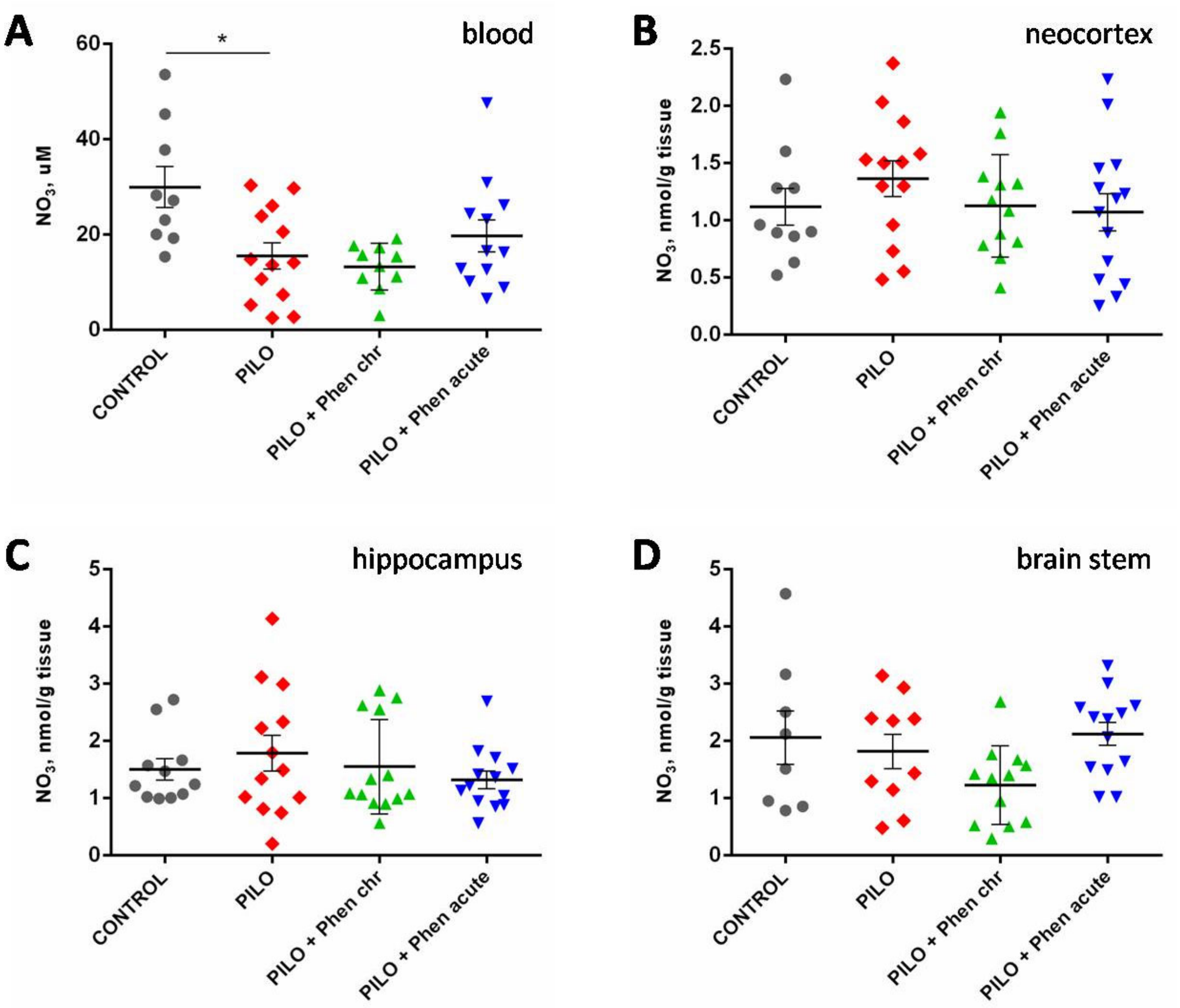

Figure S4. NO3 level in blood plasm (A) and different brain regions: neocortex (B), hippocampus (C), and brain stem (D) of rats. CONTROL - control group; PILO - pilocarpine group; Phen chr - group chronically treated with PA; Phen acute - group acutely treated with PA. * - $P<0.05$; Mann-Whitney U-test. * - $P<0.05$; Mann-Whitney U-test. Data are presented as M±SEM.

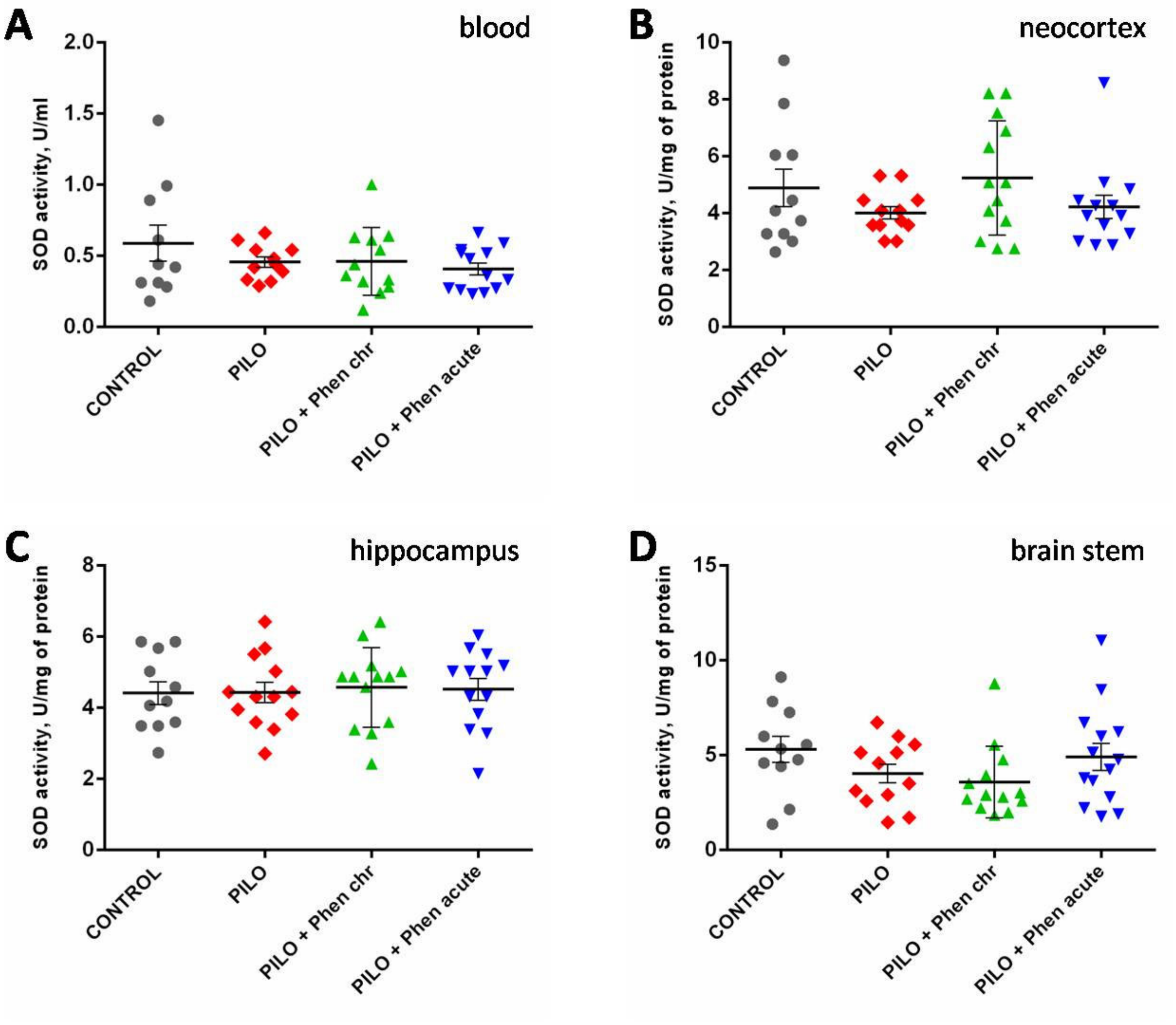

Figure S5. SOD activity in blood plasm (A) and different brain regions: neocortex (B), hippocampus (C), and brain stem (D) of rats. CONTROL - control group; PILO - pilocarpine group; Phen chr - group chronically treated with PA; Phen acute - group acutely treated with PA. Data are presented as M±SEM.

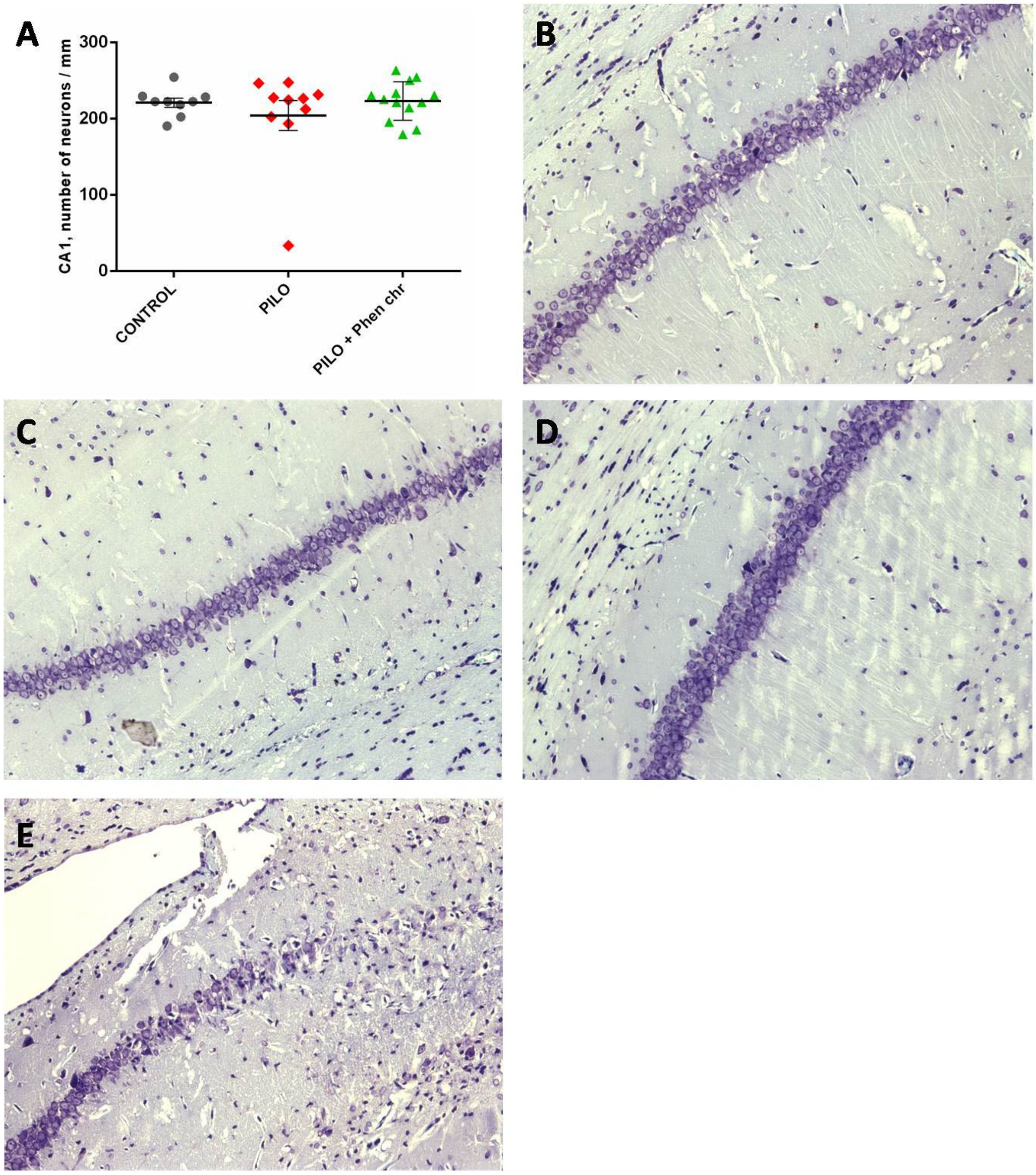

Figure S6. Number of neurons in the pyramidal layer of CA1 field. Total number (A) and representative microimages of rats from control group (B), pilocarpine SE group (C) and PA-treated pilocarpine-SE group (D). An example of prominent hippocampal sclerosis in the CA1 area of a rat from pilocarpine SE group is shown in (E). Data are presented as M±SEM. x200.

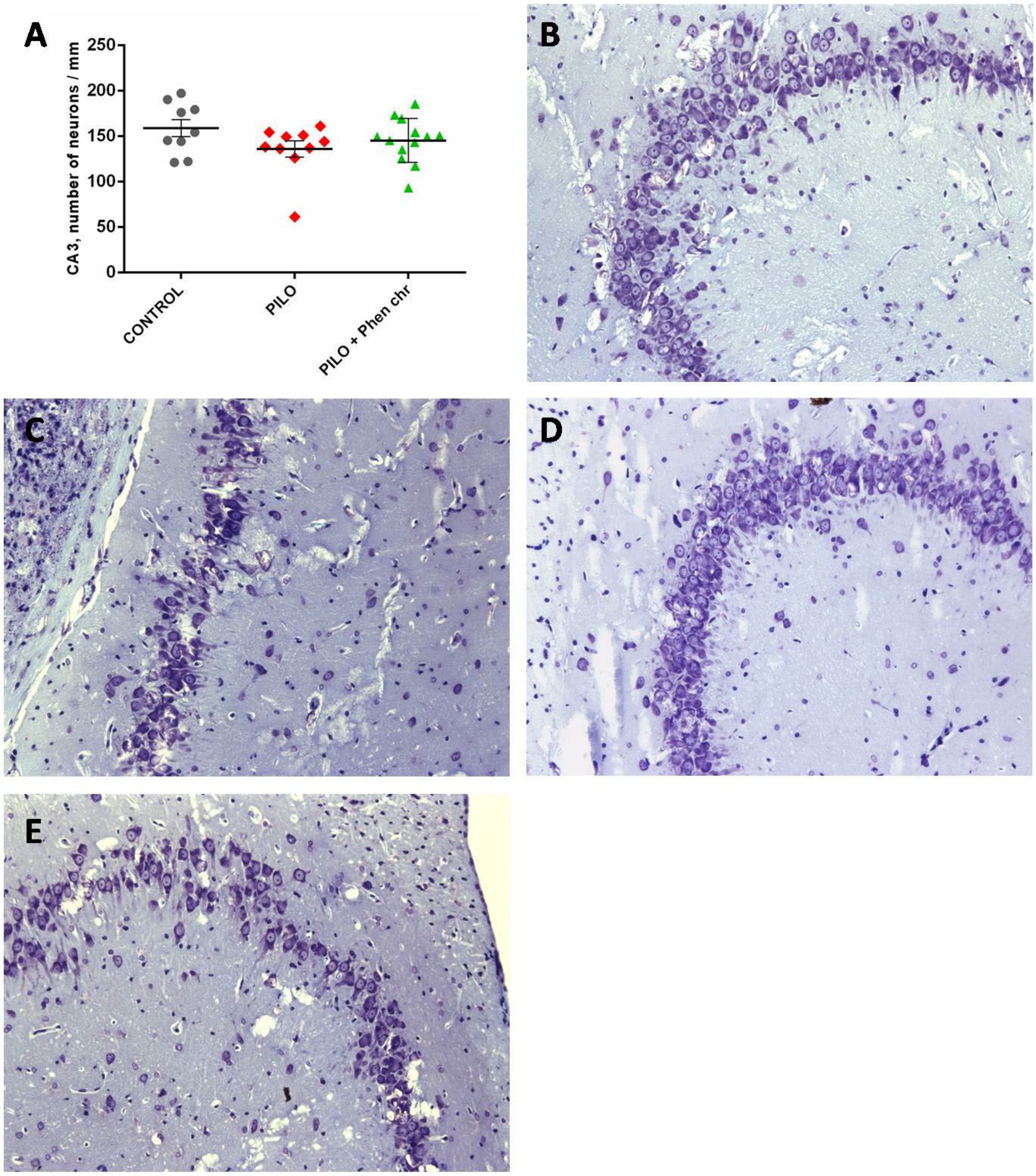

Figure S7. Number of neurons in the pyramidal layer of CA3 field. Total number (A) and representative microimages of rats from control group (B), pilocarpine SE group (C) and PA-treated pilocarpine-SE group (D). An example of prominent hippocampal sclerosis in the CA3 area of a rat from pilocarpine SE group is shown in (E). CONTROL - control group; PILO - pilocarpine group; Phen chr - group chronically treated with PA. * - $P<0.05$; Mann-Whitney U-test. Data are presented as M±SEM. x200.
